# Supplementary figures and images for: Smoking is associated with higher risk of contracting bacterial infection and pneumonia, intensive care unit admission and death
Source: PLoS One. 2024 May 9;19(5):e0302505. doi: 10.1371/journal.pone.0302505 (PMC11081217; doi:10.1371/journal.pone.0302505)

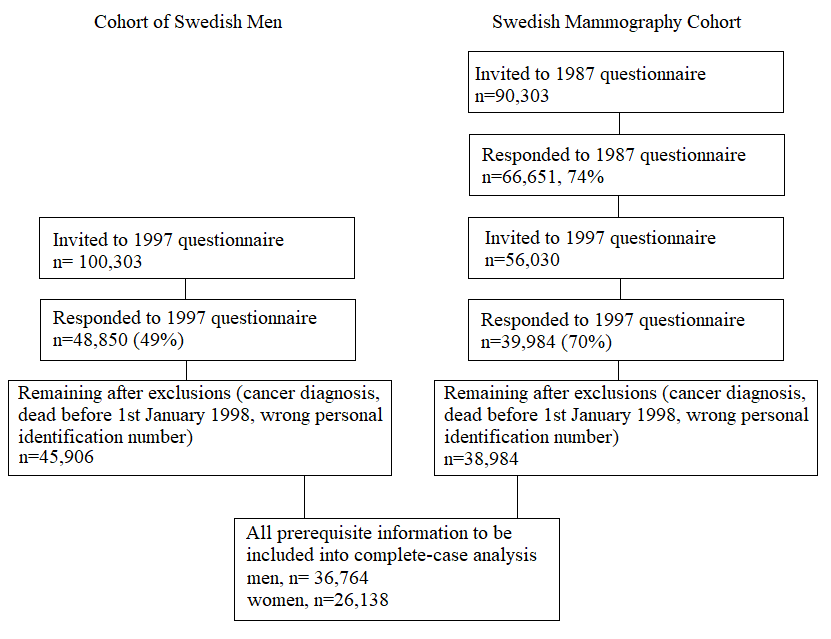

Supplement: S1 Fig — (TIF) [file pone.0302505.s003.tif]

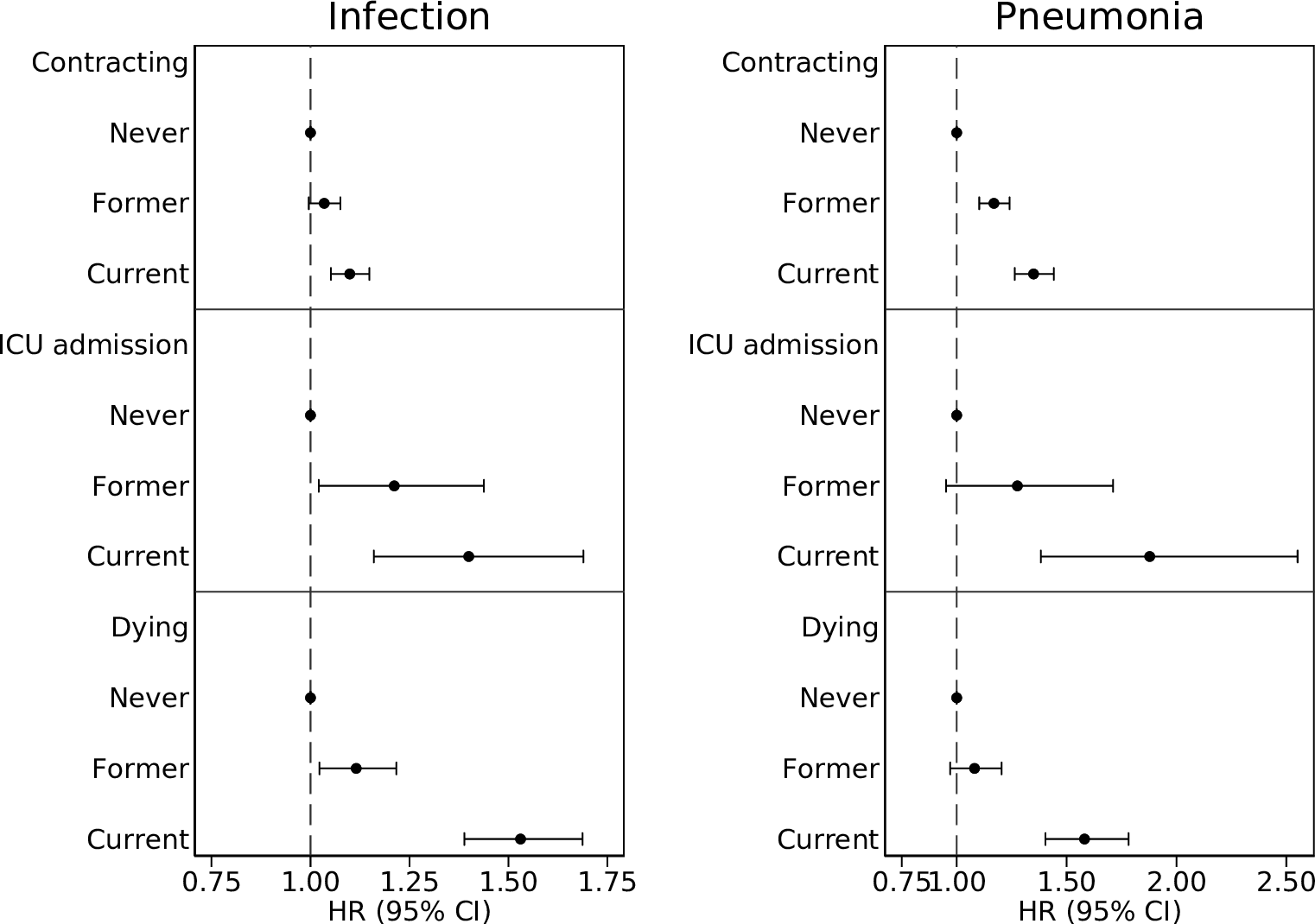

Supplement: S2 Fig — Hazard ratio (HR) and 95% confidence interval (CI) of contracting any infection or pneumonia, being admitted to an intensive care unit and dying, adjusted for age (as time scale), sex, Charlson’s weighted comorbidity index, education, marital status, exercise, walking, alcohol consumption and self-rated health. (TIF) [file pone.0302505.s004.tif]

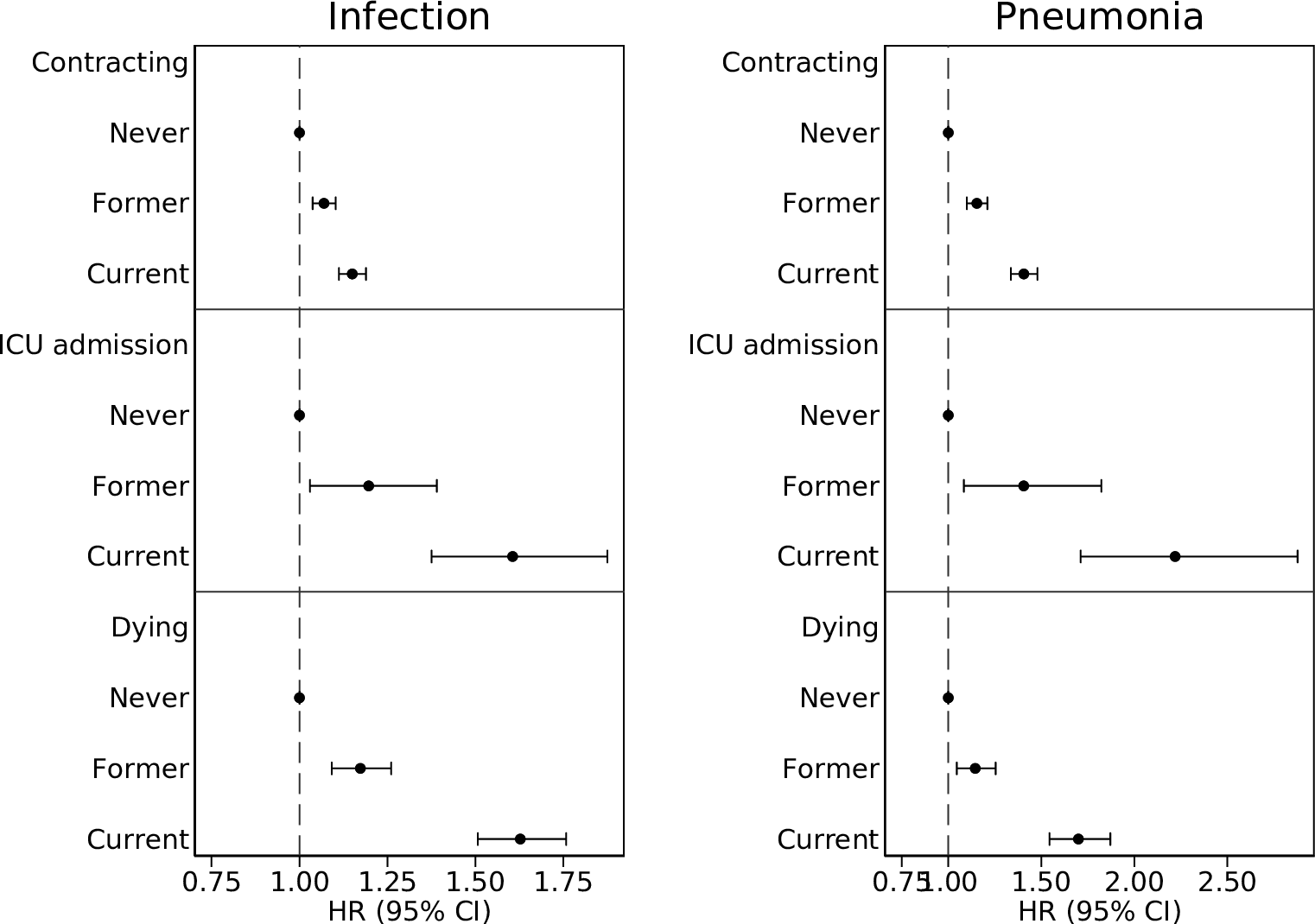

Supplement: S3 Fig — Hazard ratio (HR) and 95% confidence interval (CI) of contracting any infection or pneumonia, being admitted to an intensive care unit and dying with follow-up beginning three years after baseline, adjusted for age (as time scale), sex, Charlson’s weighted comorbidity index, education, marital status, exercise, walking and alcohol consumption. (TIF) [file pone.0302505.s005.tif]

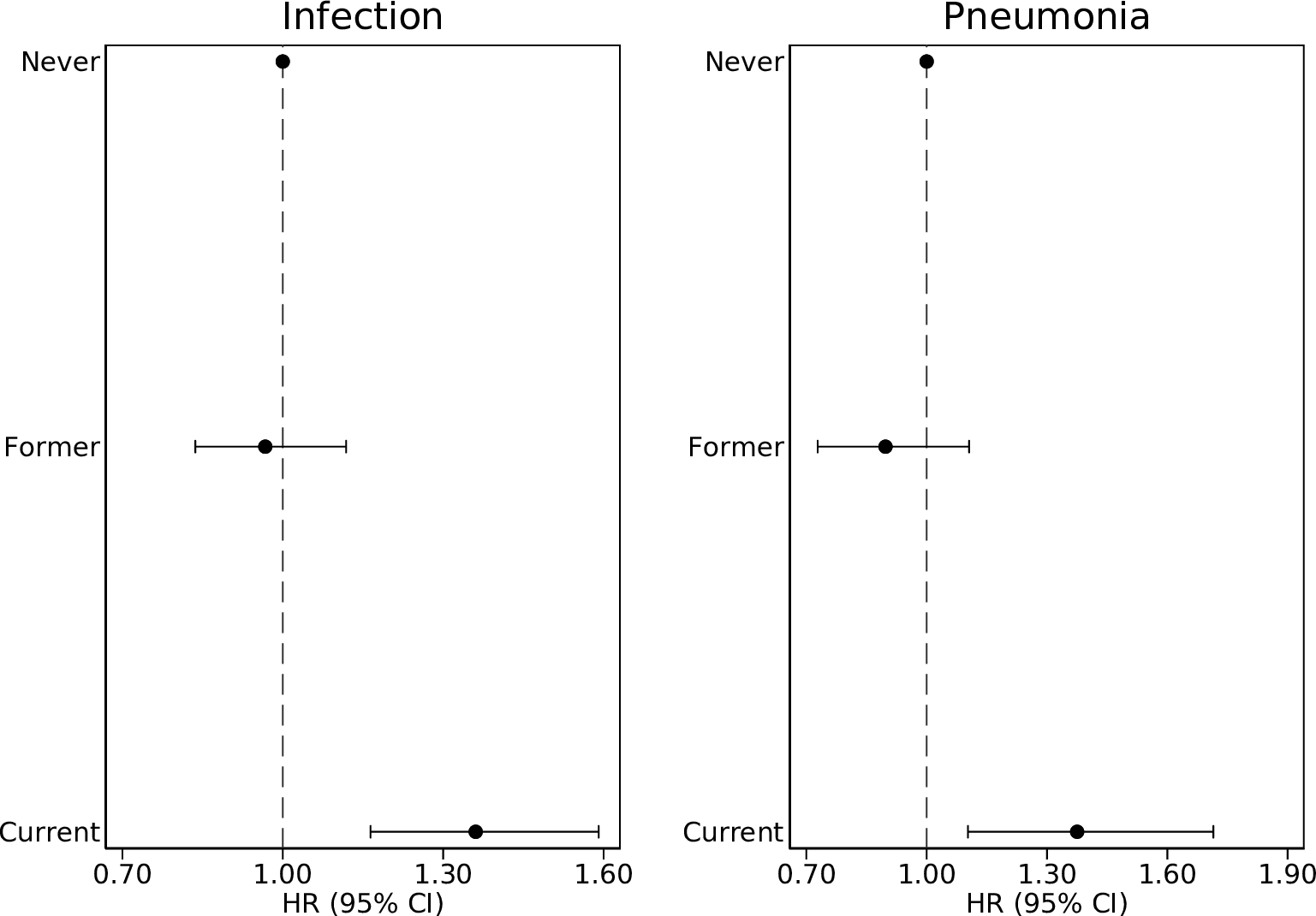

Supplement: S4 Fig — Hazard ratio (HR) and 95% confidence interval (CI) of dying in infection and pneumonia, where only the underlying cause of death is considered, adjusted for age (as time scale), sex, Charlson’s weighted comorbidity index, education, marital status, exercise, walking, and alcohol consumption. (TIF) [file pone.0302505.s006.tif]

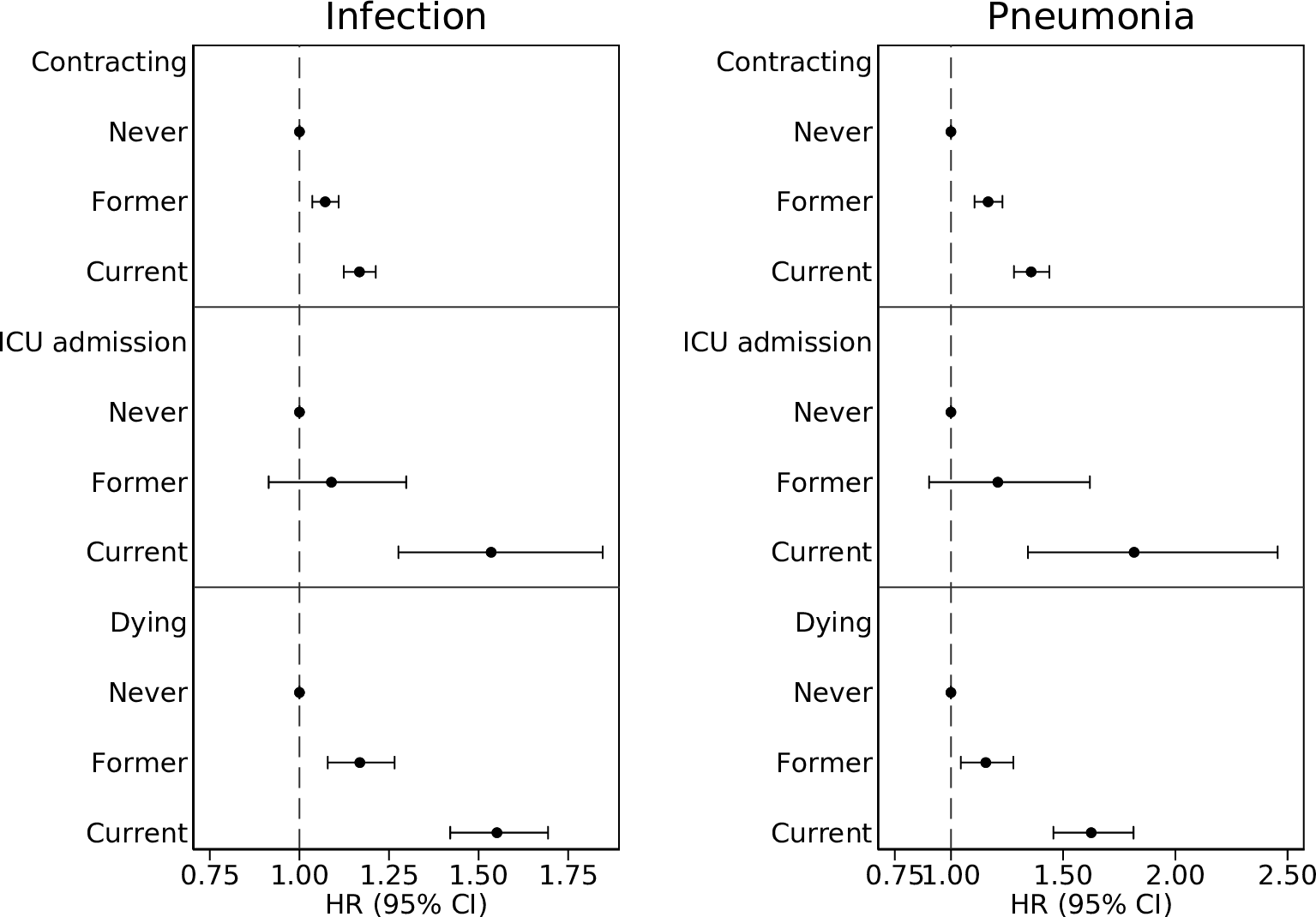

Supplement: S5 Fig — Hazard ratio (HR) and 95% confidence interval (CI) of contracting any infection or pneumonia; being admitted to an intensive care unit and dying, adjusted for age (as time scale), sex, Charlson’s weighted comorbidity index, education, marital status, exercise, walking, alcohol consumption and body mass index. (TIF) [file pone.0302505.s007.tif]

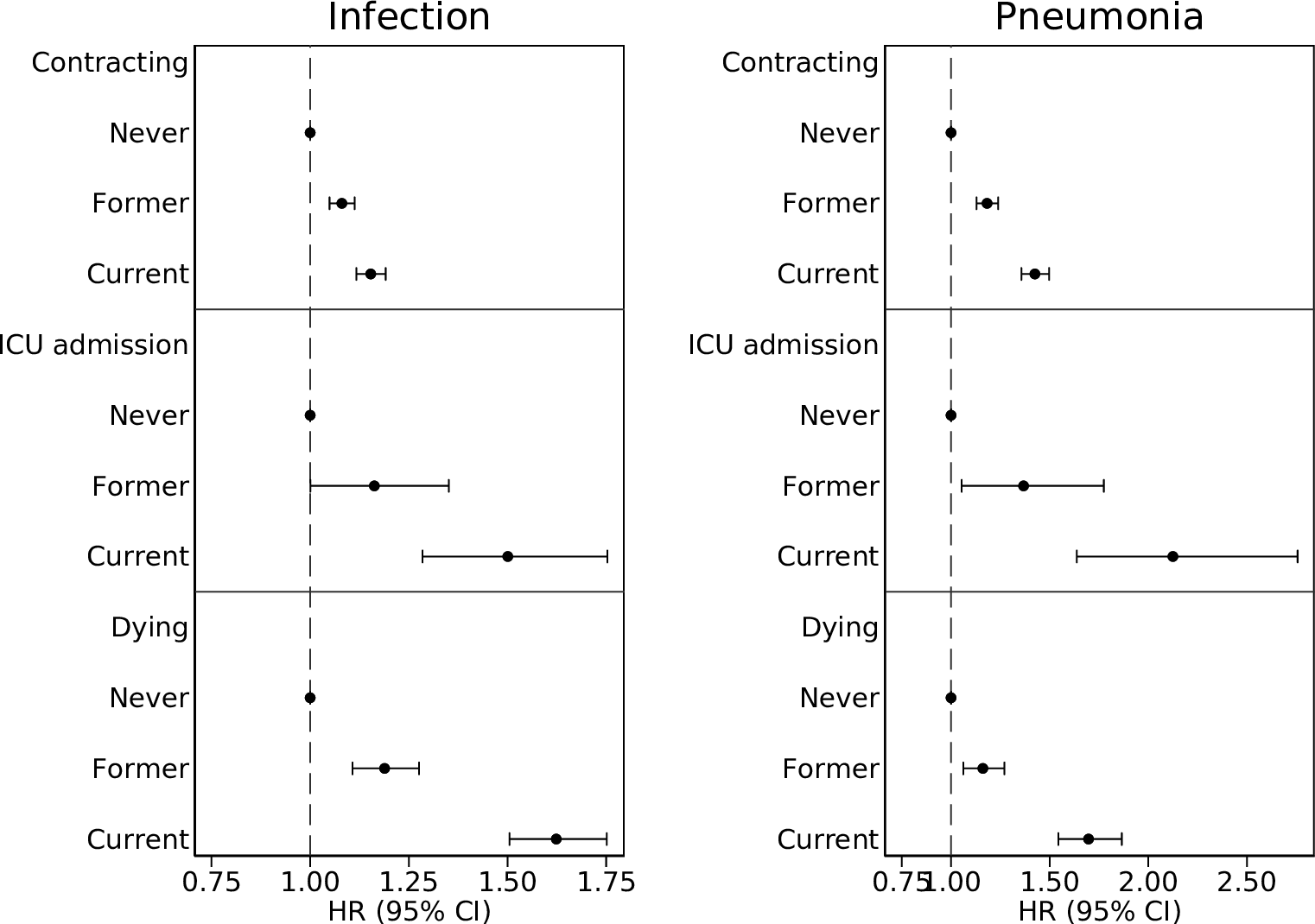

Supplement: S6 Fig — Hazard ratio (HR) and 95% confidence interval (CI) of contracting any infection or pneumonia; being admitted to an intensive care unit and dying, adjusted for time from baseline (as time scale), sex, Charlson’s weighted comorbidity index, education, marital status, exercise, walking, alcohol consumption and age. (TIF) [file pone.0302505.s008.tif]

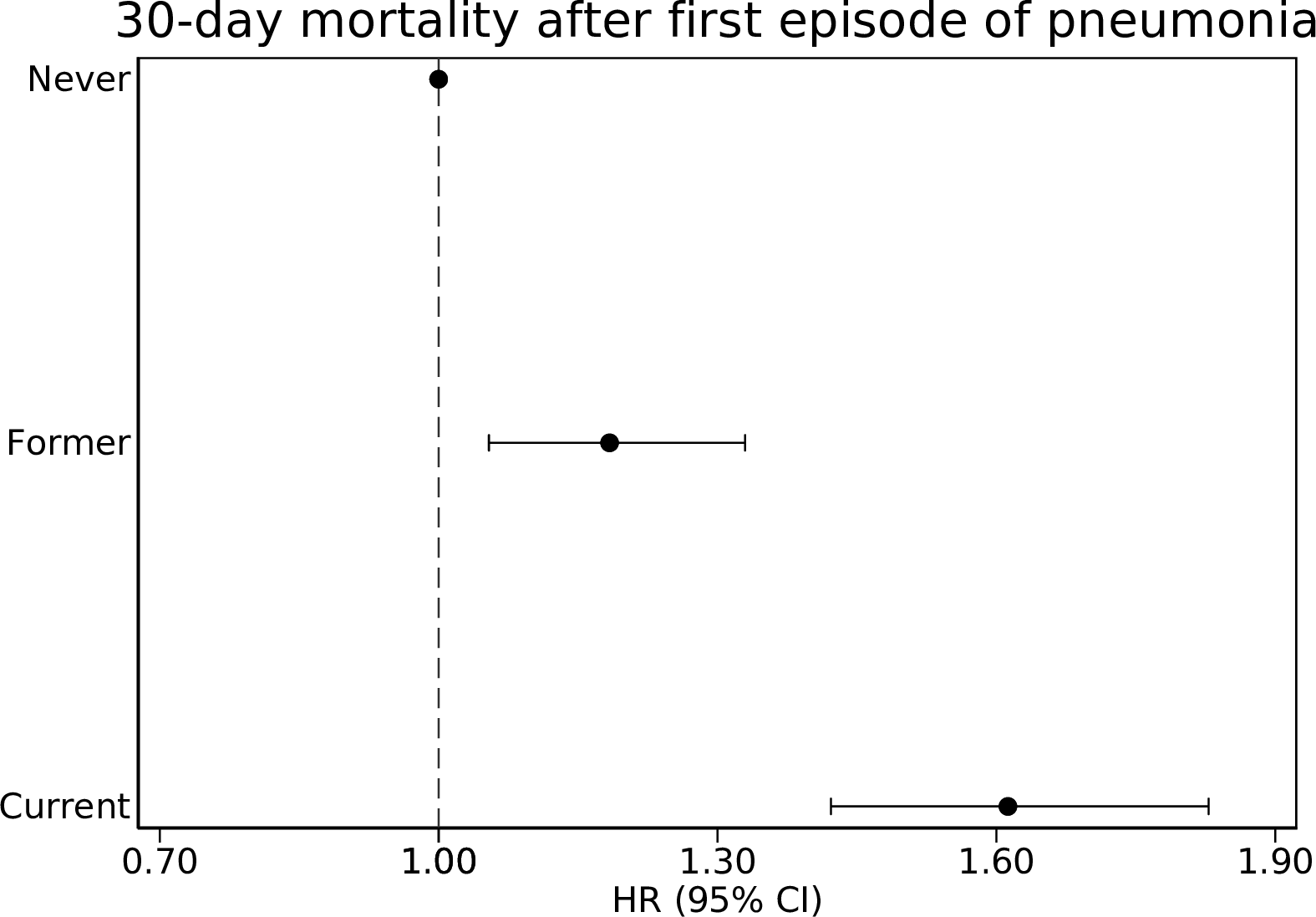

Supplement: S7 Fig — Hazard ratio (HR) and 95% confidence interval (CI) of 30-day mortality after the first diagnosis of pneumonia, adjusted for age (as time scale), sex, Charlson’s weighted comorbidity index, education, marital status, exercise, walking, and alcohol consumption. (TIF) [file pone.0302505.s009.tif]
